# Hydrodynamic Gene Delivery of CC Chemokine Binding Fc Fusion Proteins to Target Acute Vascular Inflammation *In Vivo*

**DOI:** 10.1038/srep17404

**Published:** 2015-12-01

**Authors:** Eileen McNeill, Asif J. Iqbal, Gemma E. White, Jyoti Patel, David R. Greaves, Keith M. Channon

**Affiliations:** 1 Division of Cardiovascular Medicine, British Heart Foundation Centre for Research Excellence, John Radcliffe Hospital , University of Oxford, U.K; 2 Wellcome Trust Centre for Human Genetics, Roosevelt Drive, University of Oxford, OX3 7BN, U.K; 3 Sir William Dunn School of Pathology, University of Oxford, South Parks Rd, Oxford, OX1 3RE, U.K.

## Abstract

Blockade of CC chemokines is an attractive yet under utilized therapeutic strategy. We report the *in vivo* pharmacokinetics of a broad-spectrum vaccinia virus CC chemokine binding protein (35 K) fused to human IgG1 Fc. We demonstrate that the *in vivo* efficacy of the protein can be interrogated using hydrodynamic gene delivery of a standard mammalian expression plasmid. High plasma levels of the 35 K-Fc protein are maintained for at least 14 days post gene transfer, with the protein still detectable at 5 weeks. We confirm that the protein has biological activity in acute inflammation, causing a significant reduction in monocyte recruitment during zymosan induced peritonitis. The ability of 35 K-Fc to block more complex pathologies is demonstrated using aortic digests to assess angiotensin II mediated leukocyte recruitment to the aorta. Angiotensin II causes upregulation of mCCL2 in the aorta causing the accumulation of CCR2+ cells. Peak monocyte recruitment to the aorta occurs within 3 days and this process is CC chemokine dependent, being significantly reduced by hydrodynamic delivery of 35 K-Fc.

Chronic inflammatory diseases, such as atherosclerosis and rheumatoid arthritis, are a major cause of mortality and morbidity. Cytokines and chemokines are important mediators of inflammation in these conditions, and whilst there is modulation of cytokine biology by biological therapeutics such as Etanercept, which is an Fc-fusion protein containing the TNF receptor and thus acts as a decoy receptor, licensed therapeutics targeting chemokines are very limited^1^. Although CCR5 antagonism to block HIV-entry, by Maraviroc, has been successful there are to date no licensed drugs targeting chemokines for chronic inflammatory disease^2^.

Chemokines are small soluble protein mediators that regulate leukocyte trafficking. The production of these molecules at sites of inflammation or infection drives leukocyte recruitment. When this process becomes excessive, or is inappropriately initiated, chronic inflammation and pathological tissue damage can occur^2^. Many pathogens express proteins that facilitate host immune evasion by neutralizing aspects of the host response, such as chemokine production^3^. Vaccinia virus encodes a soluble 35 kDa protein, 35 K, which binds all human and mouse CC-chemokines, typically at binding affinities greater than those for native receptor binding^4^,^5^,^6^. We have previously demonstrated that adenovirus or lentivirus mediated gene transfer of 35 K is effective at reducing atherosclerosis and vein graft disease^7^,^8^. Other groups have reported suppression of allergic inflammation in guinea pig skin and models of airway inflammation^5^,^9^. Whilst viral delivery has demonstrated the utility of anti-CC chemokine blockade in pre-clinical disease models it is unable to address issues of dose and is unlikely to be approved for human therapy if gene transfer is required.

Fc-fusions proteins are now well established as therapeutics with nine currently having FDA approval^10^. The addition of an Fc domain, human IgG1 in the case of currently licensed Fc-Fusions, has multiple benefits in construction of novel biological agents. The Fc domain is capable of binding to neonatal Fc receptors (FcRn) which are expressed on endothelial, epithelial and certain leukocyte cells. Binding of a molecule to an FcRn protects the molecule from lysosomal breakdown, instead the molecules are taken up via clathrin coated pits, whilst still bound to FcRn, which targets them to endosomal compartments allowing them to return to the cell-surface and return to circulation^11^. This property has a dramatic effect on serum half life, with human IgG having a half-life of 23 days. Indeed Abatacept, a CTLA-4 Fc fusion protein has a half-life of over 10 days^10^.

Fc-Fusion proteins also facilitate the purification of novel biologics, allowing standard antibody purification technologies to be utilized, and their detection for pharmacokinetic studies through highly specific Fc detection reagents^12^. Whilst this can facilitate design and production of novel molecules under lab-scale conditions, rather than industrial, the ability to produce large protein amounts with minimal endotoxin contamination can still be expensive and time-consuming. We have combined small-scale protein production experiments to demonstrate the single dose pharmacokinetics and systemic bioavailability of our 35 K-Fc fusion protein, with hydrodynamic delivery of the same mammalian expression plasmid used for protein production to allow efficacy of the proteins to be assessed without first needing to design multiple dosing strategies, or produce onerous amounts of highly purified fusion protein. Using hydrodynamic delivery we have demonstrated the *in vivo* utility of our CC-chemokine binding protein, mutant R89A 35 K-Fc, which we show for the first time is effective in an acute model of vascular inflammation.

## Materials and Methods

### Materials

All cell culture media and buffers were obtained from Invitrogen (UK) unless otherwise specified. All laboratory chemicals were from Sigma-Aldrich (Gillingham, UK) unless otherwise specified.

### Mice

All animal studies were conducted with ethical approval from the Local Ethical Review Committee and in accordance with the UK Home Office Animals (Scientific Procedures) Act 1986. Mice were housed in individually ventilated cages with 12 h light/dark cycle and controlled temperature (20 °C–22 °C). Standard chow (Harlan, UK) and water were available ad libitum. C57bl/J mice were purchased from Harlan UK or homozygous B6.129P2-Apoe^tm1Unc^/J, which were bred in-house, were used for experiments.

### Production of 35 K-Fc protein and Pharmacokinetics

The 35 K-Fc protein was produced by transfection of expression plasmids into 293T cells and purified by Protein A affinity, as published previously^13^. Pharmacokinetic studies were performed by ip injection of 5 μg of 35 K-Fc protein followed by peritoneal lavage and plasma sampling at harvest timepoints. Peritoneal lavage was obtained by flushing the peritoneum with 5 ml PBS/5 mM EDTA post-mortem.

### Production of 35 K-Fc plasmids for injection

35 K-Fc and mutant versions were produced in the pSecTag2(C) vector (Invitrogen, Paisley UK) as published previously^13^. DNA for injection was produced using a low-enodtoxin DNA Maxi-prep kit (Qiagen, UK) using disposable pyrogen free plasticware. Plasmid DNA was solubilized in tissue culture grade endotoxin-free water, aliquotted, and stored at −20 ^o^C. The level of endotoxin contamination was assessed using a Limulous Amoebocyte Assay (QCL-1000) (Lonza, UK).

### Hydrodynamic Delivery

Hydrodynamic delivery was performed according to established protocols^14^. Adult mice were pre-warmed to cause vasodilatation for no longer than 10 minutes, they were then anaesthetized using isoflurane. Mice were injected with 1 μg plasmid DNA diluted in 10% w/v bodyweight PBS (upto a maximum of 2 ml) in a single iv injection (27 G needle/2.5 ml syringe) over 5 secs. Mice were immediately removed from inhalation anaesthesia supply and given medical oxygen until recovered then maintained at 26–28 ^o^C for 1-2 hours until normal body temperature and activity had returned. Less than 5% animals fail to recover from anaesthesia following hydrodynamic delivery, and complications following recovery from anaesthesia are very rare <1%.

### 35 K sandwich ELISA

Maxisorb ELISA plates (Nunc, USA) were coated overnight at 4° C with vCCI (35 K) capture antibody (R&D systems) at 1 μg/ml in PBS. Plates were washed 5 × with dH2O then non-specific binding was blocked with blocking buffer (PBS/0.25% BSA/1 mM EDTA/0.05% Tween 20) for 2 hours at room temperature. Lavage samples or standards were directly incubated on the plate, or diluted in blocking buffer to within the standard curve as required and plasma samples were diluted at least 1:2 in RIPA buffer (Tris pH7.3 50 mM, NaCl 150 mM, SDS 0.1%, NP-40 1%). Samples were incubated in the ELISA plate for 2 hours, washed as above, then incubated with anti-human Fc HRP antibody (1:5000 in blocking buffer, Jackson ImmunoResearch). The plate was incubated with substrate (Sigma Fast OPD) then the reaction stopped with 3 M H_2_SO_4_.

### Bio-gel elicitation of primary mouse macrophages

C57Bl6/J (Harlan, UK) were injected intra-peritoneally (i.p.) with 1 ml of 2% Bio-gel suspended in PBS and 4 days later, mice were sacrificed and the peritoneum lavaged with 10 ml ice-cold PBS/5 mM EDTA.

### Chemotaxis bioassay

Chemotaxis was assessed using Neuroprobe ChemoTx 96 well plates (Receptor Technologies, Leamington Spa UK). Plasma, diluted 1:10 with chemotaxis buffer (RPMI/25 mM HEPES/0.5% w/v BSA) and was loaded into the lower well of the plate. RANTES/CCL5 (Peprotech EC, UK) was included in separate wells as a positive control for CC-chemokine induced migration. Bio-gel elicited primary mouse macrophages were resuspended at 5 × 10^6^ cells/ml in chemotaxis buffer and 80 μl of cell suspension (4 × 10^5^ cells) was placed on top of a filter with an 8 μm pore size. Cells were allowed to migrate for 4 hours, then unmigrated cells on top of the filter were removed by wiping with cotton buds. Migrated cells on the underside were fixed with 4% paraformaldehyde for 10 mins, then stained with DAPI for 5 mins and filters mounted onto slides. Migrated cells were assessed by fluorescence microscopy and quantified using Image Pro Plus software (Media Cybernetics Corp.). Data are expressed as migration index, i.e. fold change over the response to media alone.

### ACEA XCELLigence real-time migration

Experiments were carried out using CIM-16 well plates and an xCELLigence RTCA-DP instrument (ACEA, San Diego, USA) as previously described (Iqbal *et al.* 2013). CCL5 (2 nM) was made up in chemotaxis buffer (RPMI 1640/25 mM HEPES/0.5% (w/v) BSA) and pre-incubated with 35 K-Fc or GFP plasma (1:50 dilution) for 30 mins at 37°C. Following incubation, 160 μl was transferred into the lower wells of the CIM-16 plate. Following upper chamber attachment, the upper wells were filled with 50 μl pre-warmed chemotaxis buffer and the plate left for 10 mins at RT to pre-equilibrate. Bio-Gel elicited macrophages were resuspended to 8 × 10^6^ cells/ml in chemotaxis buffer and 50 μl cell suspension (4 × 10^5^ cells) was placed into the top wells. The assay was run over 6 h and data was collected every 5 s over the course of 4000 sweeps.

### Western Blotting for 35 K-Fc in plasma

35 K-Fc was detected in plasma by immunoprecipitation with Protein A/G agarose beads (Sigma, UK). Plasma samples were diluted 1:2 with Cell-lytic MT lysis buffer (Sigma, UK) and incubated with Protein A/G beads overnight at 4 °C with rotational mixing. The beads were washed 5 times with the lysis buffer prior to solubilisation of the bound proteins by LDS containing SDS-PAGE loading buffer (Invitrogen, UK). The resulting protein fraction was run on a western blot on a Nu-Page gel (Invitrogen) and transferred onto PVDF membrane (Amersham). The proteins were detected by blotting with anti-35 K antibodies (R&D Systems) and anti-human Fc (Jackson Labs, US) antibodies.

### RNA analysis

Tissues were excised and snap frozen. RNA was extracted using the RNeasy RNA isolation kit (Qiagen, UK) using the manufacturer’s protocol. RNA was quantified by nanodrop and cDNA was produced using the Superscript II system (Life Technologies, UK). 35 K message was measured using a custom-designed FAM-labelled Taq-man assay, designed against the 35 K sequence, using 25 ng RNA equivalent cDNA using a BioRad CFX1000 real time PCR machine. Data was normalised to GAPDH expression and then to untreated controls using the delta delta Ct method.

### Zymosan Induced Peritonitis

C57Bl6/J mice (Harlan, UK) were injected i.p. with 100 μg zymosan A (Sigma-Aldrich, Gillingham UK) diluted in 0.5 ml PBS or vehicle alone. After 16 hours the mice were sacrificed and the peritoneal cavity was lavaged with 5 ml PBS/5 mM EDTA. Peritoneal cell counts were performed using a haemocytometer and trypan blue exclusion and peritoneal exudate cells were stained with antibodies against Ly6 G (BD Biosciences, Oxford UK) and 7/4 (AbD Serotec, Kidlington UK) and analysed by flow cytometry to assess neutrophil and inflammatory monocyte recruitment.

### Ang II infusion by osmotic minipump

10–12 week old male C57bl/6J mice were anaesthetized with isoflurane by inhalation and osmotic mini-pumps (Alzet Corp, USA) delivering saline or Ang II (1 mg/kg/day) for 3 or 7 days were implanted subcutaneously.

### Aortic Flow cytometry

Descending aortae were microdissected and digested in an enzyme solution containing 60 U/ml DNase I, 60 U/ml Hyalronidase, 450 U/ml Collagenase I and 125 U/ml Collagenase XI (all enzymes from Sigma-Aldrich, Gillingham, UK) at 37 °C for one hour. A single cell suspension was prepared by passing aortic pieces through a strainer prior to subsequent flow cytometry staining. Isolated aortic cells were antibody stained for the surface markers including: PE-Cy7 conjugated anti-CD45 (BD Biosciences), total PerCP conjugated anti-CD11b (BD Biosciences), PE conjugated 7/4 (Serotech), APC conjugated anti-CCR2 (R&D systems), Pacific-blue conjugated anti-Ly-6G(BD Biosciences, UK) with appropriate isotype controls. Absolute cell counts were performed by ratio to a known quantity of calibration beads added to each sample (CaliBrite, BD Biosciences). Data was acquired using a CyAn Analyser flow cytometer (Beckman Coulter Ltd, UK) and then analyzed using FlowJo (Tree Star Inc, USA) software.

### Statistical Analysis

Between group comparisons of normally distributed measurements were assessed by Student’s t test. One-way AVOVA was used to compare more than two data groups and Dunnett’s post test was used to compare each group to a control (untreated) group. Two-way ANOVA was used to compare multiple data groups affected by two independent variables, with a Bonferroni correction to compare groups with each other. Differences were considered statistically significant at P < 0.05.

## Results

### Single Dose Pharmacokinetics of 35 K-Fc fusion protein

**Figure 1:**
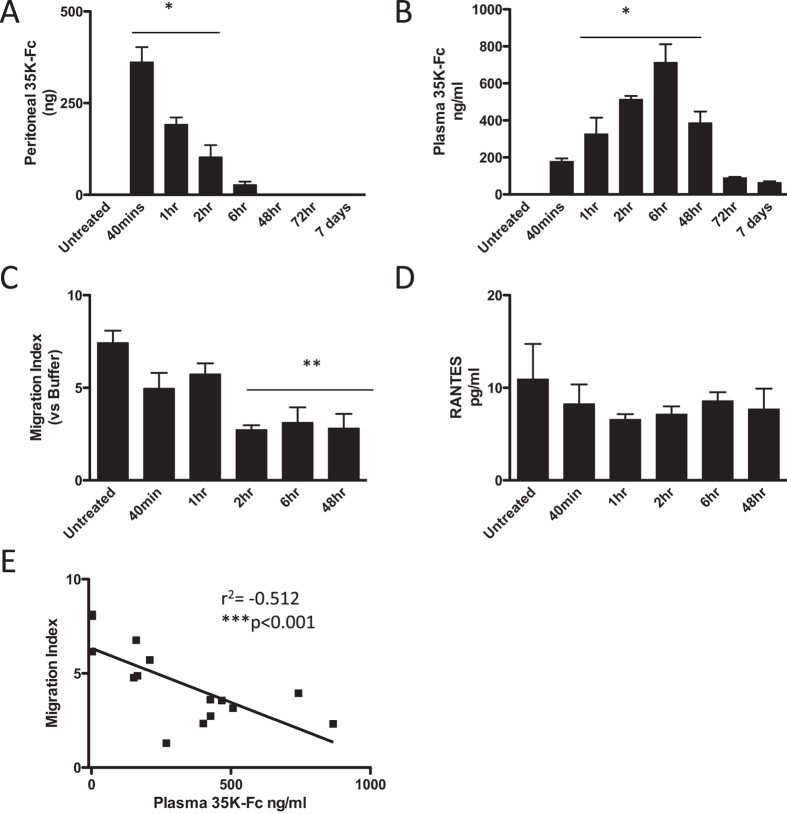
Pharmacokinetics of 35 K-Fc following ip injection. 5 μg of 35 K-Fc was injected ip into C57bl/6J mice. The peritoneal cavity was lavaged with PBS and the plasma prepared from mice harvested over 7 days following injection (n = 3 per timepoint). The concentration of 35 K-Fc in peritoneal lavage, calculated as total remaining per animal (**A**), and plasma concentration (**B**) was determined by ELISA. The bioactivity of 35 K-Fc was determined using a chemotaxis bioassay. Plasma (10%) was used as a chemoattractant in a Boyden Chamber chemotaxis assay. The number of cells migrating towards the plasma samples was calculated as a migration index normalized to the amount of random migration seen to buffer alone (**C**). The concentration of RANTES in the plasma 5 was measured by ELISA (**D**). The plasma 35 K-Fc concentration was correlated with the plasma bioactivity (**E**). n = 3 per timepoint, Statistical analysis performed by one-way ANOVA and Dunnett’s multiple comparison test. *p < 0.05, **p < 0.01, ***p < 0.001 compared to untreated. Linear correlation assessed by Spearman’s Test.

Previous studies using our 35 K-Fc fusion protein had focused on *in vitro* or local efficacy in a peritonitis model and had not addressed the systemic availability of the protein^13^. We developed an ELISA using a combination of commercially available anti-35 K and anti-human IgG antibodies to detect 35 K-Fc fusion proteins, utilizing a high salt diluent to obtain a highly specific signal in mouse plasma (harvested using either EDTA or Heparin anti-coagulants) (Supplementary Figure 1) to allow us to perform *in vivo* pharmacokinetic studies. We injected 5 ug 35 K-Fc ip into ApoE^−/−^ mice and individual animals were sacrificed either pre-injection to provide a baseline or at intervals of 40 minutes to 7 days post-injection. The peritoneal cavity was lavaged to recover protein remaining locally and plasma prepared to assess systemic availability. ApoE^−/−^ mice were used as we have previously shown that this hyperlipidemic model has elevated CC Chemokine levels in the plasma sufficient to allow the bioactivity of 35 K to be assessed in a chemotaxis assay^7^,^8^. 35 K-Fc was taken up from the peritoneum over the first 6 hours, being completely gone by 48 hours (Fig. 1A). The level of 35 K-Fc remaining in the peritoneum significantly dropped at each tested time interval after the 40 min peak, until it was no longer present at 48 hours. A reciprocal appearance of the protein in the plasma was seen, with detectable 35 K-Fc already present 40 mins after injection (Fig. 1B). The plasma levels were maintained for at least 48 hours, after which time only small amounts were detectable. The peak plasma concentration was measured 2–6 hours following ip protein delivery, with plasma protein levels being significantly lower at the other timepoints sampled. This confirmed the systemic availability of the 35 K-Fc protein. When plasma from the injected animals was used as a chemoattractant in a Boyden Chamber chemotaxis assay, using murine Biogel-elicited macrophages, we detected a significant suppression of bioactivity within 2 hours of injection and lasting for 48 hours following a single injection of 35 K-Fc (Fig. 1C). This suppression of chemokine bioactivity occurred despite no change in plasma RANTES concentration, a primary CC chemokine found in ApoE−/− plasma (Fig. 1D). Although the suppression of chemokine bioactivity seen at earlier timepoints was not significant, the plasma bioactivity was significantly correlated with plasma 35 K-Fc concentration. A highly significant correlation was observed showing a direct relationship between plasma chemokine inhibitor and reduced chemokine bioactivity in the plasma (Fig. 1E).

### Hydrodynamic delivery allows long lasting plasma bioavailability of 35 K-Fc

**Figure 2:**
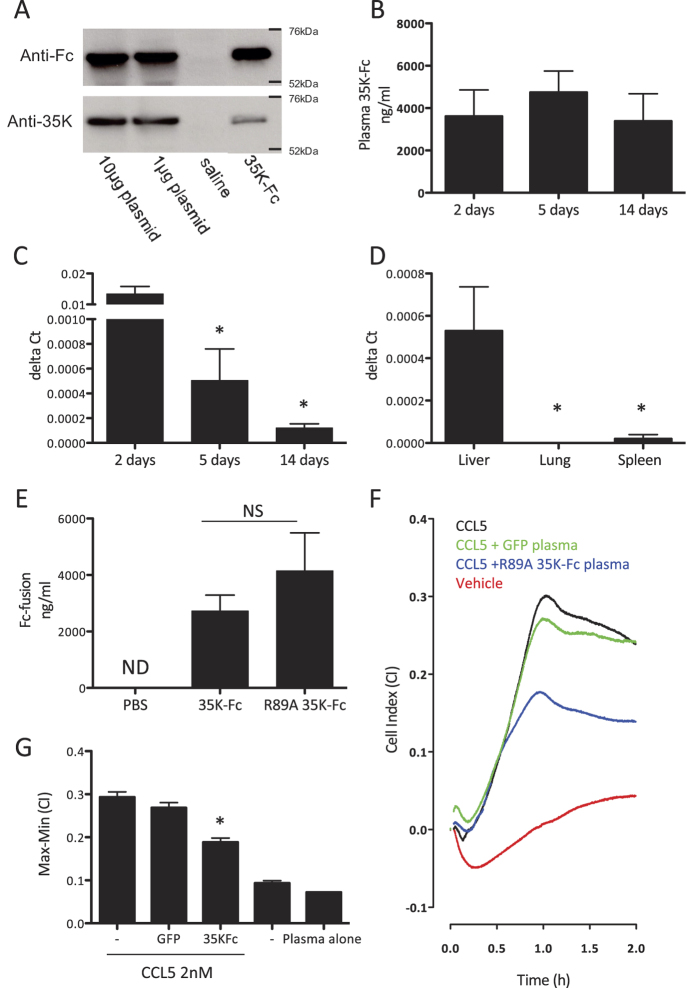
Expression of 35 K-Fc and 35 K-Fc mutants following hydrodynamic delivery. Mice were injected with 1 or 10 μg 35 K-Fc plasmid. Plasma was harvested 72 hours after injection and immunoprecipitation with protein A/G beads performed. The resulting precipitated proteins were identified by Western blot using anti-Fc and anti-35 K antibodies with purified 35 K-Fc as a positive control and saline injected animals as a negative control (**A**). Mice received hydrodynamic delivery of 1 μg 35 K-Fc plasmid, individual cohorts of mice were harvested between 2 and 14 days following injection and plasma and liver samples isolated (n = 3–5 per timepoint). Levels of 35 K-Fc in plasma were identified by ELISA (**B**) and 35 K-Fc message was assessed using specific 35 K taq-man probes in a real-time RT-PCR experiment (**C**) (*p < 0.05 by one-way ANOVA with post-tests compared to 2-day expression levels). (**D**) 35 K-Fc message was also assessed in a parallel experiment in liver, spleen and lung to assess the primary site of gene expression. To confirm that 35 K-Fc point mutants were expressed with similar efficiency 1 μg of 35 K-Fc and R89A 35 K-Fc plasmid were injected into mice and plasma harvested for ELISA after 72 hours. No significant difference in the level of plasma protein was detected (**E**). To determine the biological activity of *in vivo* expressed R89A 35 K-Fc plasma was added into a real-time chemotaxis assay. A representative trace from the RTCA-DP software showing macrophage migration towards chemoattractant (in the presence of GFP or R89A 35 K-Fc plasma at 1:50 dilution) or buffer control is shown (**F**). Quantification of the response in the xCELLigence assay showed migration towards CCL5 was significantly inhibited by the presence of R89A 35 K-Fc in the plasma samples (**G**). (n  =  3 per genotype; T-test p < 0.05 GFP vs R89A 35 K-Fc).

As the production of clinical grade, low endotoxin, protein preparations can be time-consuming and expensive we tested whether standard mammalian expression plasmids could be used to express 35 K-Fc directly *in vivo.* We prepared a low endotoxin plasmid preparation and injected 1 μg or 10 μg by hydrodynamic delivery into C57bl/6J mice and harvested plasma 72 hours later. We detected 35K-Fc in the plasma, which was confirmed to be expressed as the full molecule by Western blotting for both the Fc and 35 K portion of the fusion protein, following immunoprecipitation with Protein A-agarose beads (Fig. 2A). As similar levels of 35 K-Fc were expressed from both doses of plasmid used, we continued our studies using 1 μg of 35 K-Fc plasmid. We confirmed that hydrodynamic delivery of plasmid DNA was well tolerated by the mice by assessing weight following injection, although there was a significant drop in weight this was <2.5% (data not shown). We assessed the pharmacokinetics of 35 K-Fc expression following hydrodynamic delivery. We detected plasma 35 K-Fc at levels in excess of 3 μg/ml for at least 14 days following plasmid injection (Fig. 2B) and saw no significant drop in plasma levels between day 2 and 14. 35 K-Fc was still present in the plasma 5 weeks following injection (mean:734 ng/ml, n = 2), which is in excess of the concentration required for activity of the injected protein.

To assess to what extent the long half life of our protein in the plasma was due to continued gene expression we performed quantitative real-time RT-PCR to detect 35 K mRNA in the liver, the primary site of gene expression following hydrodynamic delivery. The levels of gene expression fall rapidly over the first 5 days but then are maintained for a number of weeks at levels sufficient to maintain a relatively constant plasma protein level (Fig. 2C). 35 K-Fc RNA expression seems to define plasma concentration as gene expression at 5 weeks after injection was only present at levels ten-fold lower than the 14 day timepoint (n = 2 data not shown), at a timepoint when plasma 35 K-Fc levels had also dropped considerably. The predominant site of expression of 35 K-Fc mRNA is the liver, as there was no detectable gene expression in the lung and barely detectable expression in the spleen (Fig. 2D). As we had previously produced a range of 35 K-Fc point-mutants including one with enhanced bioactivity (R89A 35 K-Fc), we tested whether this protein had similar expression following injection. We detected average expression levels in excess of 1 μg/ml for both versions of 35 K-Fc, with no significant difference in expression between proteins (Fig. 2E). To confirm that *in vivo* expressed 35 K-Fc has chemokine blocking activity we used a real-time chemotaxis assay to test the ability of plasma to block biogel-elicited macrophage migration towards 2 nM CCL5 (RANTES) *ex vivo* (Fig. 2F,G). Expression of R89A 35 K-Fc in the plasma, in comparison to control animals that had received a GFP expressing plasmid, was able to significantly inhibit chemotaxis to CCL5 at a dilution factor where plasma itself had no effect on cell migration.

### Expression of R89A 35 K-Fc causes inhibition of acute inflammation

**Figure 3:**
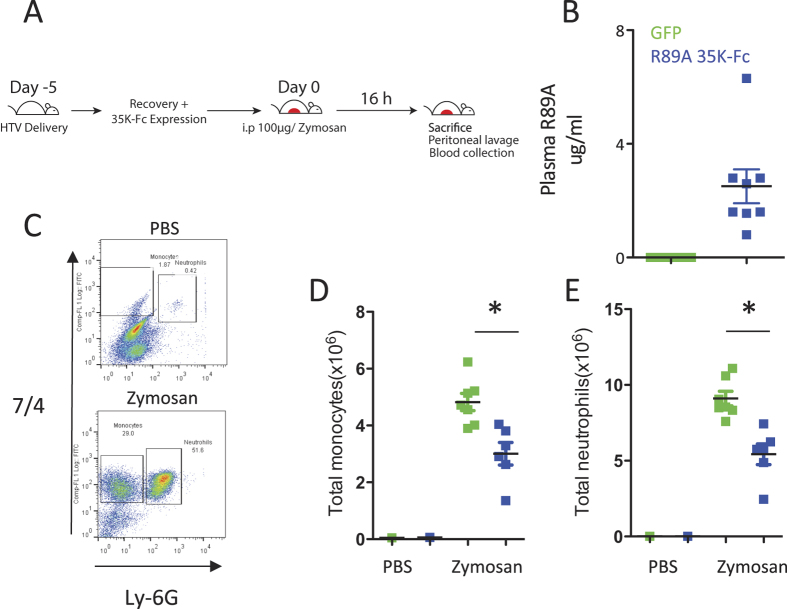
R89A 35 K-Fc expression following hydrodynamic delivery causes significant suppression of zymosan induced peritonitis. Mice were injected with 1 μg 35 K-Fc R89A 35 K-Fc plasmid or GFP control plasmid 5 days prior to injection of 100 μg zymosan ip (**A**). 16 hours after the injection of zymosan recruited cells were recovered by peritoneal lavage and plasma samples harvested for ELISA. Animals receiving R89A 35 K-Fc plasmid expressed 35 K-Fc in the plasma, whereas the protein was not detected in the GFP control animals (**B**). Leukocytes reovered from the peritoneal cavity were stained with 7/4 and Ly-6G antibodies to detect recruited monocytes and neutrophils (**C**). Injection of zymosan caused recruitment of both monocytes and neutrophils, but the number of cells recruited was significantly reduced in animals that received R89A 35 K-Fc (**D,E**). *p < 0.05. Statistical test performed by T-test.

Prior to evaluating the efficacy of R89A 35 K-Fc in *in vivo* models of inflammation we first tested whether R89A 35 K-Fc caused any confounding effects on the baseline state of the immune system. We were unable to detect any alteration of spleen:body weight ratio or on circulating leukocyte numbers in the presence of R89A 35 K-Fc expression (Supplementary Figure 2). To test whether the expression of 35 K-Fc following hydrodynamic delivery was functional and sufficient to inhibit CC chemokine mediated inflammation we performed zymosan induced peritonitis experiments in mice that had received R89A 35 K-Fc or a control GFP expression plasmid. To allow full recovery from the general anaesthesia mice were not injected with zymosan until 5 days after hydrodynamic delivery. Mice were injected with 100 ug zymosan to cause a robust recruitment of monocytes and neutrophils to the peritoneum (Fig. 3A). 16 hours after injection with zymosan or saline mice were culled and peritoneal lavage performed and plasma samples prepared. R89 35 K-Fc expression was confirmed in the mice by plasma ELISA and all mice had detectable protein levels with an average of 2.5 μg/ml (Fig. 3B). The cells harvested by lavage were stained to identify monocytes and neutrophils and the recruitment of cells enumerated (Fig. 3C). A small number of saline injected animals were analysed to confirm the expected lack of cells without zymosan injection. We observed a significant reduction (38–40%) in the number of monocytes and neutrophils recruited in animals expressing R89A 35 K-Fc compared to control GFP animals (Fig. 3D,E).

### Aortic monocyte recruitment in Angiotensin II infused animals is CC chemokine dependent

**Figure 4:**
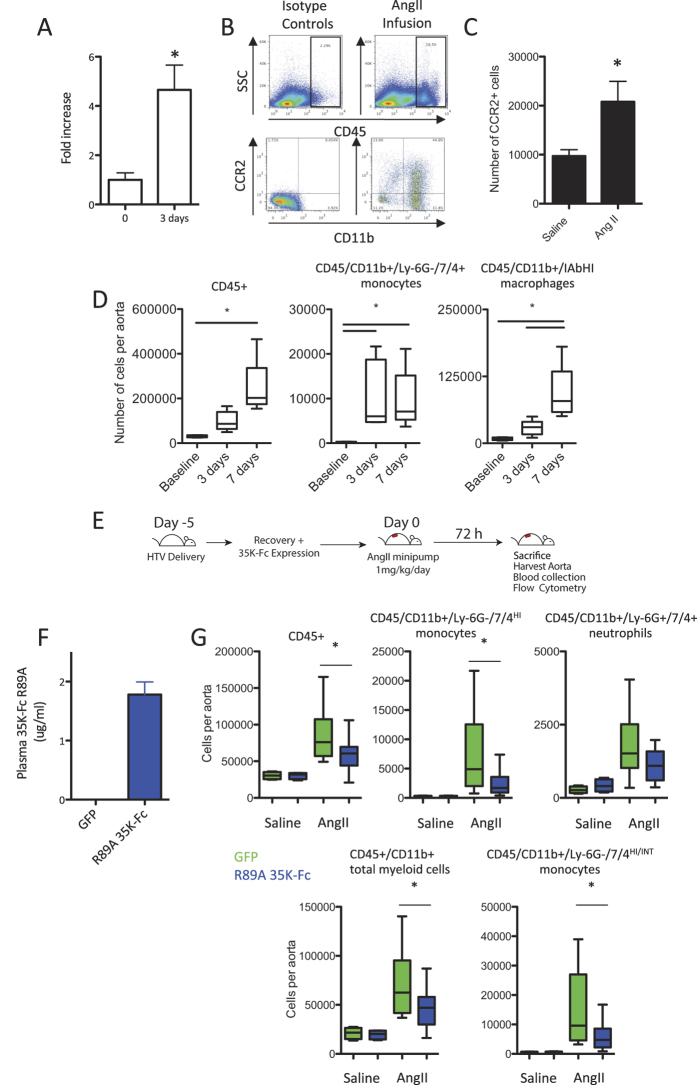
AngII infusion causes a CC-chemokine dependent recruitment of monocytes to the aorta that is inhibited by R89A 35 K-Fc. Mice were treated with AngII at 1 mg/kg/day by subcutaneous osmotic minipump. Aorta were harvested from mice following 3 days of infusion and RNA was prepared. A significant induction of CCL2 was measured in the mice following AngII infusion compared to baseline uninfused control animals (n = 4) (**A**). Digestion of isolated aortas showed CCR2+/CD11b+ leukocytes within the aorta (**B**) these cells were significantly increased in number following 7 days of Ang II infusion, vs saline control animals (n = 4–8) (**C**). Aortas harvested over a timecourse of AngII infusion showed a progressive recruitment of leuckoytes and in particular monocytes and macrophages to the aorta (n = 5–8) (**D**). To assess the efficacy of R89A 35K-Fc in inhibiting this recruitment, mice were injected with 1 μg R89A 35 K-Fc plasmid or GFP control plasmid 5 days prior to implanatation of an AngII minipump. Mice were sacrificed after 72 hours of AngII treatment (**E**). Analysis of plasma confirmed expression of 35 K-Fc by ELISA (**F**). Analysis of the recruited cells showed a significant blunting of the recruitment of total CD45+ leukocytes, CD11b+ myeloid cells and monocytes (inflammatory 7/4^HI^ and total 7/4^HI/INT^) to the aorta, but no significant effect on neutrophil recruitment (**G**) (n = 12–13 AngII, n = 4 Saline). *p < 0.05. Statistical testing was performed by T-test (two groups only) or One-way ANOVA with Dunnett’s post testing.

Having shown that R89A 35 K-Fc protein expression following hydrodynamic delivery was systemically available and effective in a model of peritoneal inflammation we next decided to test whether the protein would be effective in another, more complex pathology that has been reported to be CC chemokine dependent^15^. We infused C57bl/6J animals with Angiotensin II (Ang II) at 1 mg/kg/day and performed gene expression studies on the aorta from mice 3 after 3 days of AngII infusion compared to un-infused controls. We showed a significant induction of aortic CCL2 expression following Ang II treatment (Fig. 4A). To assess whether this expression results in an increased recruitment of leukocytes bearing the CCL2 receptor, CCR2, we isolated aortas from untreated and Ang II infused mice. We performed enzymatic digests and used flow cytometry and a cell counting protocol to enumerate the number of CCR2+ cells in the aorta (Fig. 4B). In keeping with the increased expression of CCL2 we observed that the number of CCR2+ myeloid (CD11b+) cells resident in the aorta 7 days after initiation of AngII infusion was increased (Fig. 4C). We next quantified the recruitment of leukocytes (defined as CD45+) in response to AngII infusion in more detail. Leukocytes were recruited to the aorta over the 7 days of the experiment, with a significant recruitment of monocytes within 3 days, a number that remained unchanged at 7 days (Fig. 4D). In contrast only small numbers of cells exhibiting macrophage markers were recruited to the aorta within 3 days, but this number was significantly increased at 7 days, presumably as the recruited monocytes differentiated to become macrophages.

To assess whether R89A 35 K-Fc is sufficient to block monocyte recruitment to the aorta we again performed hydrodynamic delivery 5 days prior to the induction of inflammation (Fig. 4E). Osmotic minipumps were implanted to infuse the mice with 1 mg/kg/day AngII for three days following which mice were culled and aortas digested to assess leukocyte numbers and plasma R89A 35 K-Fc levels measured. We found that R89A 35 K-Fc expression was maintained at plasma concentrations above 1 μg/ml at the harvest time point, 8 days following injection (Fig. 4F). R89A 35 K-Fc expression was sufficient to reduce total leukocyte numbers in AngII infused aortas by 32% and in particular significantly inhibited total myeloid cell (CD45+/CD11b+) and monocyte recruitment (both the inflammatory 7/4^HI^ and total 7/4^HI^ and ^INT^ populations). The number of recruited monocytes was reduced by 64%, whereas no significant effect on neutrophil recruitment was observed (Fig. 4G and Supplementary Figure 3).

## Discussion

We have demonstrated the *in vivo* utility of our 35 K-Fc reagents for probing the role of CC chemokines in pathology. We have demonstrated that systemic broad-spectrum CC-chemokine inhibition is effective in reducing myeloid cell recruitment in response to zymosan peritonitis. We have also demonstrated that 35 K-Fc can block the arrival of monocytes in the early phase of AngII driven vascular inflammation, and that a preferential blockade of monocyte, rather than neutrophil recruitment is observed. Furthermore, we have demonstrated the utility of our platform for testing novel Fc-fusion proteins without the need for production of large quantities of purified protein. Our previous work showed that 35 K-Fc mutants had altered abilities to block CC chemokines *in vitro* and locally *in vivo* when delivered to the site of inflammation. We have now demonstrated that 35 K-Fc proteins have good single dose pharmacokinetics, and also that they have systemic efficacy in established CC-chemokine dependent pathologies.

Fc-fusion technology is a powerful tool to facilitate new drug design. Whilst this technology has many positive aspects in drug production and serum half-life, it also has limitations^12^. Addition of the Fc portion increases the size of the molecule, which may impact tissue penetration. The construction of the fusion protein may also create unanticipated neo-antigens that render the Fc-fusion protein more immunogenic than either portion alone^10^. Early testing and mitigation of these potential limitations during development, as well as demonstration of proof of principle activity in disease models, can be facilitated by our hydrodynamic delivery strategy. The ability to produce one plasmid from which both *in vitro* protein production and long-term *in vivo* expression can be achieved allows effective streamlining of the development process. Whilst we have used a standard mammalian plasmid, with protein expression driven by a modified CMV promoter, further investment in this platform would benefit from plasmid engineering to select a promoter that would be more effective in the liver, the main site of protein expression following hydrodynamic delivery, as the CMV promoter has been reported to be silenced by the liver^16^. Recent publications addressing the optimization of gene expression from the liver have highlighted changes to a CMV driver plasmid, such as removing CpG repeats and adding a human elongation factor 1α promoter with CMV promoter elements, can increase *in vivo* expression markedly, without compromising *in vitro* gene expression^17^. Using a more sophisticated plasmid should allow testing of novel proteins in more chronic pathologies requiring gene expression over a number of weeks or months, something that can be prohibitively expensive in early proof of concept studies.

The development of aortic aneurysm and dissections are increasingly understood to be reliant on vascular inflammation. The recruitment and local activation of inflammatory cells is key to the development of these pathologies with knockout of CCR2 globally, or within the hematopoietic compartment by bone marrow chimerism, being protective from aneurysm formation in both normo- and hyper-lipidemic mice^18^,^19^. Indeed more selective assessment of local macrophage recruitment to the aorta was potently reduced with a related reduction in vascular hypertrophy^20^,^21^. The recruitment of CCR2 expressing monocytes into the aortas of CCR2−/− mice has been shown to reverse protection, causing increased IL-6 production and increasing the occurrence of aortic dissection^15^. The strong link of this pathology with CC chemokines makes this a suitable model to test our novel anti-CC chemokine therapy. We have demonstrated that a lower dose of 1 mg/kg/day Ang II is sufficient to cause acute monocyte recruitment and accumulation of macrophages within days of treatment, despite being lower than the 3 mg/kg/day dose required to cause acute aortic dissection in C57bl/6J mice. This dose allows the biology of AngII-induced vascular inflammation to be probed without confounding secondary effects of aortic dissection, allowing study of the mechanisms that predispose the aorta to dissection^22^. The kinetics of this accumulation of inflammatory monocytes is similar to the kinetics observed in the more inflammation prone ApoE−/− model^23^. Our novel CC-chemokine inhibitor caused a significant inhibition of monocyte and mature myeloid cell recruitment to the aorta, in keeping with the published role of CCL2. This confirms that expression following hydrodynamic plasmid delivery is sufficient to cause inhibition of disease, providing powerful proof of concept data without the need for design and testing of multiple dosing schedules.

We observed a slightly variable effect of CC chemokine inhibition on neutrophil recruitment in the two models tested. Neutrophil recruitment by chemokines is highly dependent on CXC chemokines with CXCR2−/− mice or mice with a conditional induced CXCR2 deficiency showing reduced neutrophil recruitment in models of peritoneal inflammation^24^,^25^. A significant effect was seen on neutrophil recruitment in the peritonitis model. This was not observed in our previous studies where 35 K-Fc was delivered locally to the peritoneum during ongoing inflammation. In contrast in the current study 35 K-Fc was present at the onset of inflammation. Our data indicate either that CC chemokines have a more pronounced role in neutrophil recruitment when a higher dose of zymosan is injected, or that the inhibition of early arriving CC chemokine dependent cell populations has a secondary effect on neutrophil recruitment. We have recently demonstrated that CCR2−/− mice also show a significant reduction in the number of neutrophils recruited in the zymosan peritonitis model, although this effect is of a much small magnitude to that seen on monocyte recruitment^26^. When we assess neutrophil recruitment in our vascular inflammation model we do not see a significant effect on neutrophil recruitment, although a trend to a decrease is observed. There is evidence that AngII induces potent neutrophil chemokines *in vivo,* such as CXCL1 and C5a, which may account for the lesser effect of 35 K-Fc on neutrophil recruitment in response to AngII^27^,^28^. This would be in keeping with a more minor or complex role of CC chemokines in neutrophil recruitment in this model.

The ability to obtain proof of biological activity, without production of large amounts of protein, allows more rapid progress to be achieved and, in principle, a greater variety of proteins to be screened for activity. A similar chemokine inhibiting Fc fusion protein based on vaccinia virus 35 K (vCCI) tested in the collagen-induced arthiritis model required multiple doses of 200 μg to be delivered to show a biological effect^29^. Over the course of the experiment this required 2 mg of protein to be delivered per mouse. This dosing regime was associated with a loss of efficacy and the generation of a strong anti-body response to vCCI-Fc within 14 days of beginning treatment^29^. We have expressed 35 K by lenti-virus and have observed functional inhibition of disease for at least 3 months, a far longer period than achieved with vCCI-Fc^7^. If production of an Fc-fusion protein does cause an enhanced immune response, potentially through the creation of neo-antigens, this is again something that can be screened for using modified plasmids, without the need for multi-dosing experiments using modified proteins.

Potent immune modulatory therapeutics like TNFα inhibitors, show potent inhibition of immune diseases, such as rheumatoid arthiritis but their efficacy can be lost over time requiring a second line of therapy^30^. The potency of cytokines like TNFα and the CC chemokine family in fundamental immune functions such as control of infection, leads to real concerns about the cost to benefit relationship of positive effects on disease vs compromising the ability of the body to control infections and cancers. However, the success of the TNFα-Fc therapies demonstrates that well monitored patients can derive great benefit from anti-cytokine therapies. The association of CC chemokines with so many chronic conditions such as atherosclerosis, arthritis and gastro-intestinal disease indicates that CC-chemokine inhibitors, such as our 35 K-Fc mutants may have utility both as experimental tools to probe the role of this class of molecule and as potential therapeutic agents^2^.

## Additional Information

**How to cite this article**: McNeill, E. *et al.* Hydrodynamic Gene Delivery of CC Chemokine Binding Fc Fusion Proteins to Target Acute Vascular Inflammation *In Vivo. Sci. Rep.* **5**, 17404; doi: 10.1038/srep17404 (2015).

## Supplementary Material

Supplementary Information
