# Supplementary material for: Hydrodynamic Gene Delivery of CC Chemokine Binding Fc Fusion Proteins to Target Acute Vascular Inflammation In Vivo
Source: Sci Rep. 2015 Dec 1;5:17404. doi: 10.1038/srep17404 (PMC4664965; doi:10.1038/srep17404)
Supplement: Supplementary Information [file srep17404-s1.pdf]

Hydrodynamic Gene Delivery of CC Chemokine Binding Fc Fusion Proteins to Target Acute Vascular Inflammation *In Vivo*

Supplementary Data

Eileen McNeill[1,2], Asif J. Iqbal[3,], Gemma E. White[3], Jyoti Patel[1,2], David R. Greaves[3], Keith M. Channon[1,2]

[1] Division of Cardiovascular Medicine, British Heart Foundation Centre for Research Excellence, John Radcliffe Hospital,
[2] Wellcome Trust Centre for Human Genetics, and
[3] Sir William Dunn School of Pathology, University of Oxford

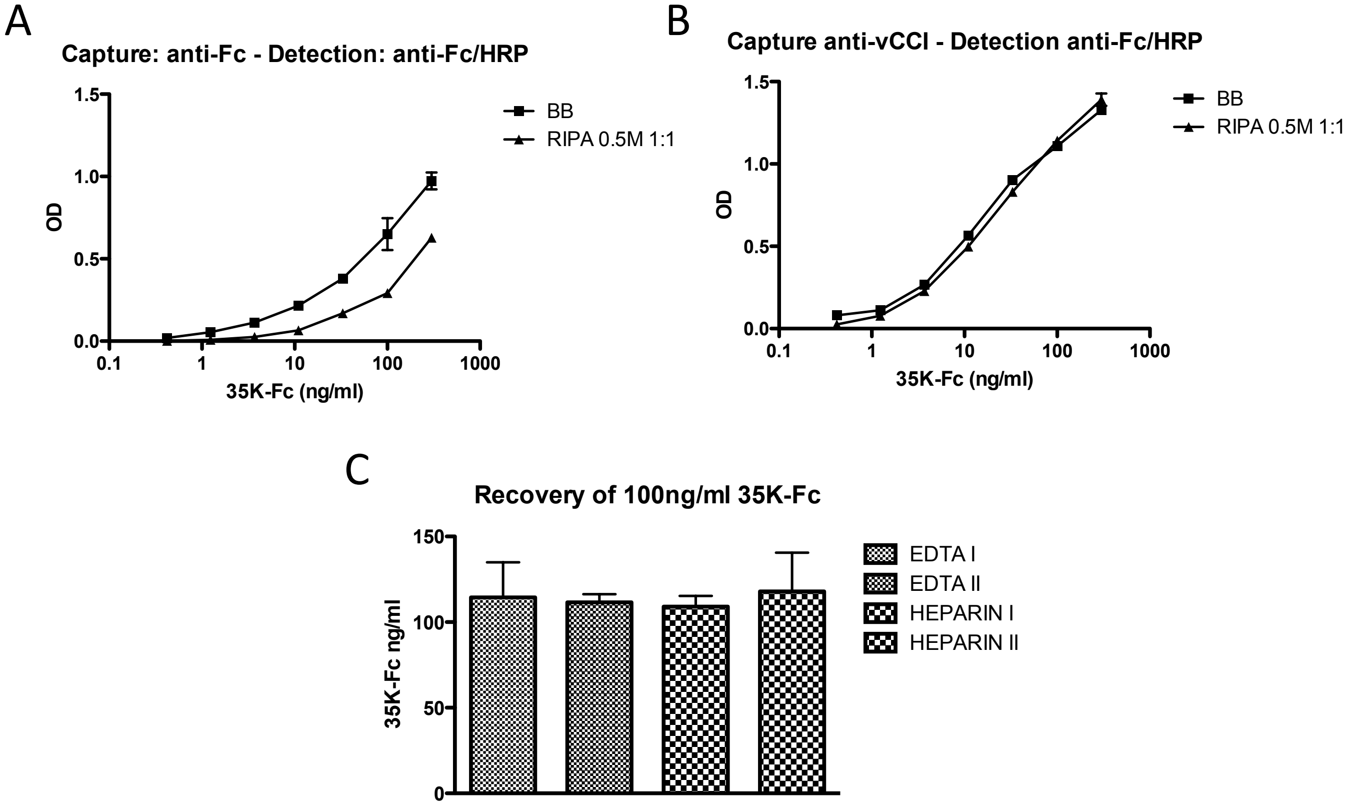

Supplementary Figure 1: Detection of 35K-Fc in plasma by ELISA. 35K-Fc protein was diluted into either standard blocking buffer or plasma diluted 1:1 with RIPA buffer and detected by capture with either anti-Fc or anti-vCCI followed by detection with anti-Fc/HRPconjugated antibody. Recovery of 100ng of 35K-Fc spiked into plasma harvested into EDTA or Heparin coated tubes was confirmed to be 100% when dilute 1:1 in RIPA buffer and subjected to ELISA using anti-vCCI capture antibody.

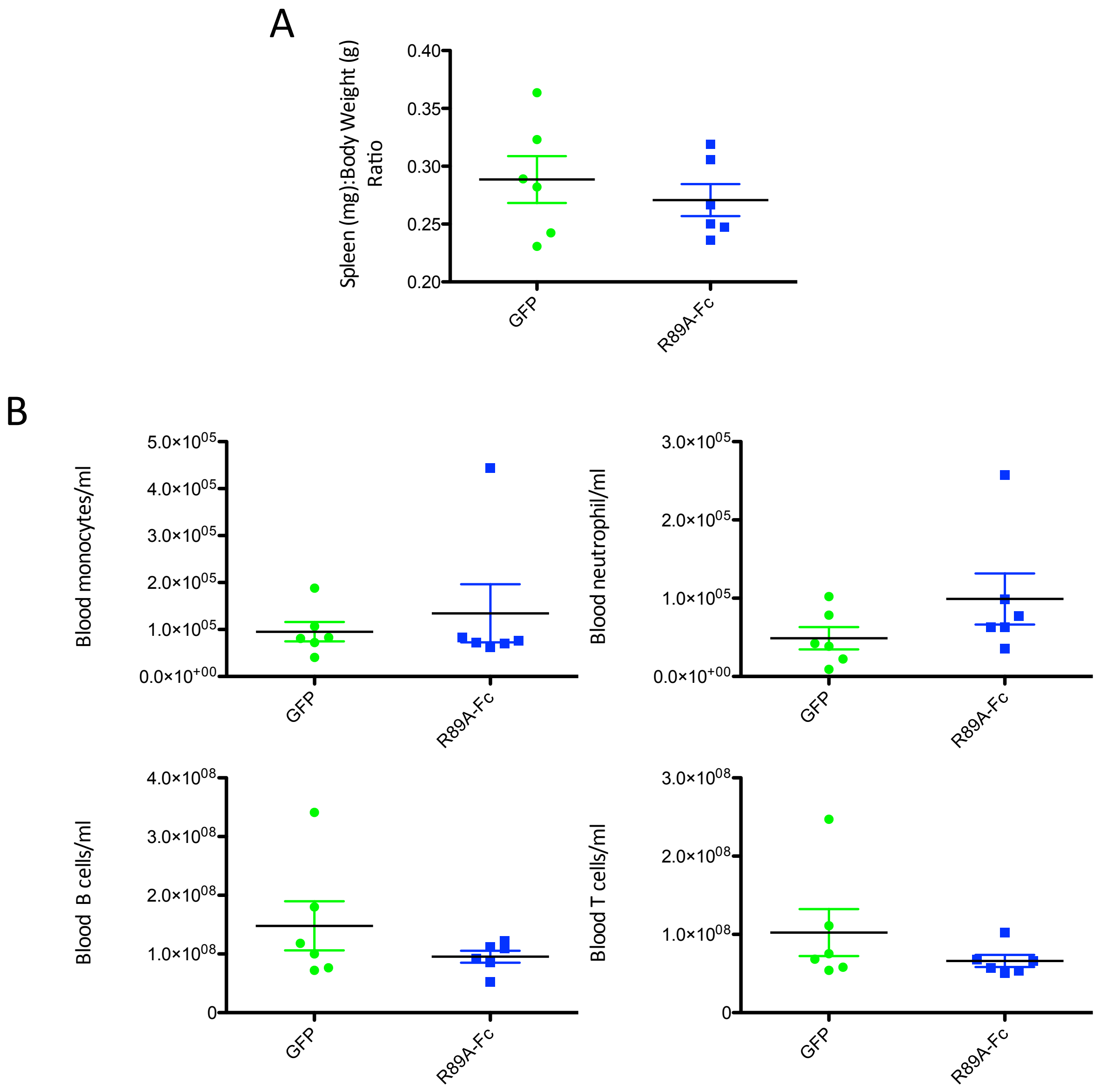

Supplementary Figure 3: Effect of R89A 35K-Fc on immune parameters. Mice were culled 5 days following hydrodynamic delivery of R89A 35K-Fc or GFP plasmids. (A) Spleen:Body weight ratio was calculated and no significant difference between the groups was observed. **(B)** Blood samples were taken and were stained with CD45/CD11b/Ly-6G and 7/4 to identifiy neutrophils ($CD45^+/CD11b^+/7/4^{HI}/Ly\text{-}6G^+$) and inflammatory monocytes ($CD45^+/CD11b^+/7/4^{HI}/Ly\text{-}6G^-$). A parallel set of samples were stained withCD45, B220 and CD3 to identify B cells ($CD45^+/B220^+$) and T cells ($CD45^+/CD3^+$). Cells were enumerated using an absolute count protocol as described in the methods section.

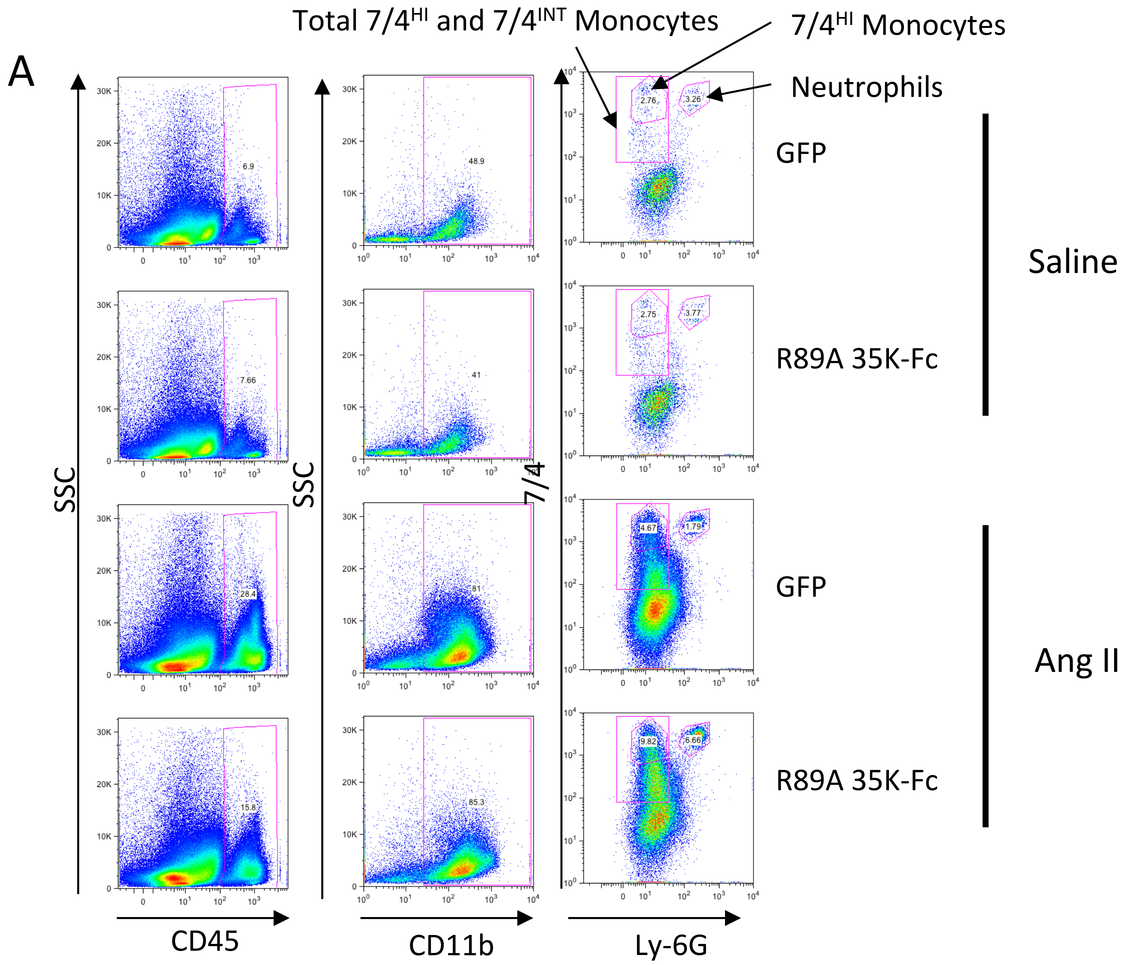

Supplementary Figure 2: Recruitment of monocytes and neutrophils to the aorta in response to 1mg/kg/day AngII. Single cell suspensions prepared from AngII infused aortas were stained with anti-CD45, CD11b, 7/4 and Ly-6G antibodies and analysed by flow cytometry. Representative dot plots showing the recruitment of cells in response to AngII are shown and were used to quantify the cell recruitment in Figure 4.
